# New Library-Based Methods for Nontargeted Compound Identification by GC-EI-MS

**DOI:** 10.1021/jasms.4c00451

**Published:** 2025-01-14

**Authors:** Deborah F. McGlynn, Lindsay D. Yee, H. Martin Garraffo, Lewis Y. Geer, Tytus D. Mak, Yuri A. Mirokhin, Dmitrii V. Tchekhovskoi, Coty N. Jen, Allen H. Goldstein, Anthony J. Kearsley, Stephen E. Stein

**Affiliations:** † Applied and Computational Mathematics Division, National Institute of Standards and Technology, Gaithersburg, Maryland 20899, United States; ‡ Department of Environmental Science, Policy, & Management, University of California at Berkeley, Berkeley California 94720, United States; § Biomolecular Measurement Division, National Institute of Standards and Technology, Gaithersburg, Maryland 20899-8362, United States

## Abstract

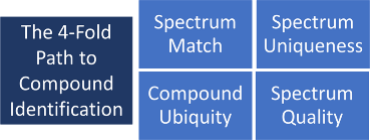

While gas chromatography mass spectrometry (GC-MS) has long been used to identify compounds in complex mixtures, this process is often subjective and time-consuming and leaves a large fraction of seemingly good-quality spectra unidentified. In this work, we describe a set of new mass spectral library-based methods to assist compound identification in complex mixtures. These methods employ mass spectral uniqueness and compound ubiquity of library entries alongside noise reduction and automated comparison of retention indices to library compounds. As a test data set, we used a publicly available electron ionization mass spectrometry data set consisting of 4833 spectra of particulate organic compounds emitted by combustion of wildland fuels. In the present work, spectra in this data set were first identified using the NIST 2023 EI-MS Library and associated batch process identification software (NIST MS PepSearch) using retention-index corrected Identity Search scoring. Resulting identifications and related information were then employed to parametrize other factors that correlate with identification. A method for identifying compounds absent from but related to those present in mass spectral libraries using the Hybrid Similarity Search is illustrated. Nevertheless, some 90% of the spectra remain unidentified. Through comparison of unidentified to identified mass spectra in this data set, a new simple measure, namely median relative abundance, was developed for evaluating the likelihood of identification.

## 1 Introduction

Analysis of complex samples of organic compounds commonly employs gas chromatography electron ionization mass spectrometry (GC-EI-MS) due to its ability to separate, identify, and quantify these compounds. Following elution of a compound from the GCcolumn, it is ionized and fragmented by electron ionization mass spectrometry, generally at an energy of 70 eV. Under these conditions, a given compound can produce a reproducible spectrum of fragment ions, and this “fragmentogram” then serves as its “fingerprint” for identification. These spectra are then tentatively identified by matching to spectra in reference mass spectral libraries. Observed retention times in the GC column, after conversion to the Kovàts retention index (RI), are commonly used to confirm the initial identification. These RIs depend on the specific column type, which are often categorized as “polar”, “nonpolar”, or “semistandard nonpolar” and are most often determined relative to *n*-alkane retention times acquired in a separate run. When an unknown compound is compared to a library spectrum, a measure of spectral similarity is computed, which then serves to form a “hit list” sorted by score, with the highest scoring library spectrum at the top. This measure is variously called a match factor or match score and is commonly calculated using a modified cosine similarity function.^1^ Another key measure is the uniqueness of a spectrum, as measured by differences in match factor, which can be expressed as the probability of being correct, assuming the matching compound is in the library.^2^

However, despite steady improvements in measurement methods and enhanced mass spectral libraries, this process of identification is commonly laborious, subjective, and often successful for only a small fraction of the spectra in complex mixtures. In nontargeted analysis, multiple factors, including chromatographic resolution, spectral contamination, variability, and similarity of spectra within certain compound classes, make the direct matching to reference spectra not always a reliable means of compound identification. For confident identification in these studies, chemical analysts must use additional information such as chromatographic retention indices (RIs) to distinguish between compounds with similar mass spectra. In the present work, RIs refer to widely used Kovàts retention indices. This is facilitated by the availability of RI values for all compounds in the NIST 2023 EI library,^3^ including reliable AI estimates^4^ for compounds without measured values for widely used semistandard nonpolar columns. Differences between experimental and reference RIs may be directly employed for match factor score penalization. Use of RIs for confirmation of identity is essential in complex mixtures, a task that is generally performed manually after spectrum matching.

In this work, we illustrate the application of newly developed, largely automated methods for increasing the confidence of nontarget compound identification in complex mixtures using nonspectral information derived from the library. This was developed using data from the NOAA program “Fire Influence on Regional and Global Environments Experiment” (FIREX), available from the University of California, Berkeley FIREX data set.^5−7^ Specifically, this is a GC-EI-MS database in NIST MS format containing mass spectral and RI information for compounds emitted by burning wildland fuels during the 2016 FIREX-AQ campaign at the Missoula Fire Science Laboratory. Besides making direct use of the recent availability of RI for all compounds in the NIST 2023 EI-MS library, other information derived from the library was employed to develop additional measures that assist identification. These measures are based on three basic factors: spectrum uniqueness, compound ubiquity, and spurious peak rejection by reverse search scoring. Each of these metrics is described further in Methods and illustrated in Results and Discussion. Scoring corrections are suggested for each factor and employed to create a composite score for hit list creation. Next, we illustrate the application of the Hybrid Similarity Search^8−10^ for identifying compounds not represented in the library whose molecular mass may be estimated from their spectra with the aid of their RI. Finally, to investigate why a large fraction of spectra could not be identified, we compared features of identified and unidentified spectra in the FIREX data set. In this analysis, we found that median peak abundances, a measure of the dynamic range of abundances, correlated strongly with spectrum identifiability with lower values (higher dynamic range) favoring identifiable spectra. Evidence for this simple yet surprising finding is discussed in detail.

## 2 Methods

### Data Set

The FIREX mass spectral data set contains information on particulate organic compounds emitted from the burning of various fuel combinations (vegetation) typically consumed during western US wildland fires at the US Forest Service Fire Sciences Laboratory in Missoula, MT. The data set contains 4833 mass spectra with Kovàts RI of compounds separated by using two-dimensional gas chromatography coupled with electron ionization time-of-flight mass spectrometry (GCxGC EI-MS) that utilized online trimethylsilyl (TMS) derivatization. In GCxGC (2D-GC), compounds are separated in the first dimension by volatility in a semistandard nonpolar column, followed with separation in the second dimension by polarity in a polar column. Analysis by 2D-GC served to aid identification of compounds that coelute in the first dimension. Of the 4833 separated compounds, 148 spectra were reported as identified based on authentic standards, RI, and the NIST14 EI-MS library.^3^ Spectra included in this collection were evaluated and selected based on their quality and uniqueness. Also, peaks from perfluoromethyldecalin (PFMD), an internal standard in these experiments, were removed from about 38% of FIREX spectra prior to searching based on the presence of its characteristic peaks (69, 131, 243, and 293 Da).

### Identification by Spectrum/Retention Index Scoring

Spectra were analyzed by matching the NIST23 EI-MS library (NIST/EPA/NIH 2023) using the NIST MSPepSearch v0.9.7.1 program,^11^ which generates simple tab-separated output text files. Individual spectrum examination was done using the MS Search v3.0 user interface program.^12^ These two programs generate identical scores. Retention index score adjustment was applied when absolute value differences between the measured retention indices and reference RI values in the NIST23 library (*dRI*) exceeded threshold values. This threshold was set at 15 Kovàts units with the penalty for identification outside of this range set at the “average” level in the NIST MS Search program, which reduced the score by 50 × (*dRI* – 15)/15 score units, where the maximum reported score is 999 (see NIST MS PepSearch documentation^11^). The column type was set as semistandard nonpolar, the primary column of the FIREX studies and the one used for RI determination. Note that the time spent in the polar column second dimension was short, only ∼2.3 s, corresponding roughly to 1.3 Kovàts retention units, hence without significant effect on RI matching. The median absolute RI deviation for high scoring compounds (>750) was 9 RI units, with first and third quartile values of 5 and 16. Unless stated otherwise, all reported scores use the Identity Search mode with match score penalization according to differences in the query (FIREX library) and reference mass spectra.

### AIRI (Artificial Intelligence Retention Index)

To ensure that RI values were available for all compounds in the NIST library, a reliable method for the estimation of these values from chemical structures was developed. Using empirical semistandard nonpolar column RI data for the146k compounds in the NIST 2023 Retention Index Library for training, RIs for all compounds in the NIST 2023 EI Library were estimated using an AI method presented in Geer et al.^4^ These values are called Artificial Intelligence Retention Indices, or AIRI. AIRI values were assigned for all compounds in the EI library including some 153 thousand of the 347 thousand compounds without experimental values. After comparison with experimental values, median and mean errors were reported in this paper as 8.0 and 15.1 retention units, respectively, and used for score adjustment when experimental data were not available.

### Spectrum Contamination

Three methods are available for reducing the contribution of spurious peaks to spectrum match scores.

1 The “Reverse Identity Search” score rejects contaminant peaks in a query spectrum when absent from a matching library spectrum. This is referred to simply as Reverse Search in this paper.
2 Since high mass peaks are weighted by the square of the mass in the standard Identity Search,^1,13^ high mass contamination peaks can significantly penalize scores. The Similarity and Hybrid Searches do not use this weighting, so they inherently minimize these effects.
3 Abundance thresholding may be used for spectra with pervasive low abundance noise throughout the spectrum. This crude method can lead to the loss of high-mass ions often needed to distinguish isomers and establish the molecular mass.

In this work, due to the presence of significant contaminant peaks, especially at higher masses, we find that combining the Reverse Search score with the Identity Search score, which are always generated together, is the best method for identification in the presence of contaminant peaks.

### Compound Filtering

FIREX samples were derivatized using *N*-methyl-*N*-(trimethylsilyl)trifluoroacetamide (MSTFA), a process where hydrogen atoms on hydroxy and amino groups are replaced with trimethylsilyl (TMS) groups. In view of the high efficiency of this process, the relative concentration of molecules with free hydroxy or amino groups should generally be relatively small. However, even with retention index constraints, incorrect high scoring library entries can contain free hydroxy or amino groups. This is a particular problem for aliphatic alcohols, which can readily lose their hydroxyl groups prior to fragmentation. For example, scores, spectra, and retention indices are almost identical for 1-eicosanol (C_20_H_42_O, score 861, *RI* = 2282, and *dRI* of 12 RI units) and 1-tricosene (C_23_H_46_, score 860, *RI* = 2291, and *dRI* of 3 units), both of which appeared in the same hit list for a query spectrum with an *RI* of 2294. The TMS derivative of the former compound was, in fact, identified 60 *RI* units later, and an identification of 1-eicosanol would almost certainly be incorrect. While removal of underivatized alcohols is currently a task for analysts, this was done in a filtering step before hit list construction. In the future, this filtering step will be done using structural information in the NIST library. In this process, partially derivatized compounds with at least one TMS group are not filtered.

### Identification Probability

For each identification, the NIST program reports an identification “Probability” along with each Identity score under the assumption that the spectrum of the compound that produced the spectrum is in the library.^2^ It is based on the idea that larger differences in scores imply larger differences in relative likelihoods of being correct. These relative likelihoods are computed using parameters derived from results of searches using alternate high-quality spectra of compounds known to be in the library. This depends on three factors—the uniqueness of the spectrum among library spectra, the likelihood that a false positive will appear at that score, and the quality of the spectrum. To illustrate the significance of this quantity, consider the case where the two top hits have the same score. In this case, each has an equal and 50% maximum likelihood of being correct regardless of the spectrum match score.

### Compound Ubiquity

A key factor in establishing the confidence of any identification is its “prior probability”, or the likelihood, prior to analysis, that the compound is present and identifiable in the sample.^14^ Presently, this must be determined by an analyst with expertise in the analysis of related materials. However, a general measure of the commonality, or ubiquity, of a compound is given in NIST23 as the “DBs” value, referred to here as the Compound Ubiquity Index (CUI). This is simply the number of 58 diverse collections of chemicals that cite the compound (see Supplementary Table 1).

### Hybrid Similarity Search (HSS)

For compounds not confidently identified by the RI-corrected Identity Search and whose molecular mass may be estimated, the Hybrid Similarity Search method offers a means of finding closely related compounds in the library and possibly enabling a tentative structural identification to be made.^9,10^ In addition to conventional peak matching, this method attempts to match neutral loss peaks whose mass is defined as the difference between its mass and the mass of the molecular ion. This is done by logically shifting the masses of the library peaks by the difference in molecular mass of the query and library compound, a quantity termed DeltaMass. This method can identify compounds closely related to those in the library when structural differences do not significantly alter the fragmentation mechanisms. However, manual confirmation is required to assign plausible chemical formula differences from DeltaMass, to ensure consistency with measured RI and confirm that shifted mass peaks are consistent with fragmentation rules. Moreover, HSS hit lists often have many more high scoring entries to be examined than does the Identity Search (see Supplementary Figures S1a–c). In fact, it can generate multiple “correct” identifications. Some identifications can be straightforward. For example, identifications with DeltaMass values that are multiples of methylene (14 Da), suggest insertion or deletion of one or more CH_2_ units. Generally speaking, each addition or subtraction of a methylene group adds or subtracts 100 Kovàts units (each CH_2_ in a linear alkane contributes 100 to the Kovàts index). Other DeltaMass values are possible by replacement of one group with another. For example, replacement of a methylene with an ether oxygen leads to a DeltaMass of 2, as would changing a double bond to a single bond. Simple changes in structure can often be quickly estimated using “Group Additivity” methods.^15^

### Scoring

Since scores are bounded by 0 and 999, in this work the correction to the initial match factor (or score) *MF1*, is made by modifying it by *MFcorr* using eq 1
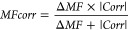
1where *Corr* (aka “correction”) is the sum of two score adjustments described later (for CUI and Probability). When *Corr* is positive, Δ*MF* is the difference between *MF1* and the maximum of 999 and *MFcorr* is added to *MF1* to create the final score. When *Corr* is negative, Δ*MF* is the difference between *MF1* and the minimum, zero, or simply *MF1*. In this case, *MFcorr* is subtracted from *MF1* to derive the final score. This only occurs for CUI = 0. Examples of use are given in the next section.

## 3 Results and Discussion

Mass spectral library-based identifications of GC-EI-MS spectra begin with the generation of a “nearest neighbor” hit list of library compounds sorted by decreasing similarity to the query spectrum. Initial identifications unconfirmed by analysts have been called ‘Tentatively Identified Compounds’ by the EPA.^16^ In practice, using retention index data along with an understanding of the composition of the sample under study and the quality of the data, the analyst is responsible for confirming each identification. This process can employ score cutoffs and subjective reasoning in making an identification. The work presented below is intended to assist in this process.

### Reported Identifications and RI-Corrected Identity Scores

**Figure 1:**
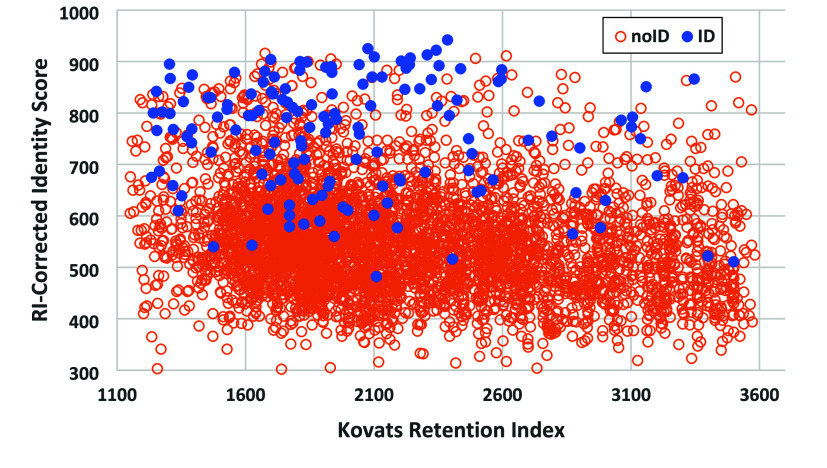
Retention-index corrected Identity Scores versus Kovàts retention index for the FIREX data set from analysis of the NIST 2023 EI Library using the NIST MSPepsearch program v. 0.9.7.1. Reported identifications from the NIST 2014 Library are solid blue circles, and unidentified spectra are empty orange circles.

The present analysis begins with an examination of the results of searching the FIREX data by means of the 2023 NIST EI library and software. Scores for spectra in the FIREX data set versus retention index, with reported identifications based on the 2014 NIST EI Library, are shown in blue in Figure 1. Scores were computed using the Identity Search method with retention index correction, as described earlier.

Distributions of the FIREX spectra depicted in Figure 1 are shown in Table 1, including distributions of Reverse Identity Search scores with RI-correction and spectra with the PFMD calibrant removed.

**Table 1:** RI-Corrected Score Distributions for FIREX Spectra in Figure 1^a^

Scores	All	Identified	Reverse Score	PFMD Removed
>900	14	9	56	0
>800–900	155	52	363	1
>700–800	296	40	651	50
>600–700	905	33	1456	152
>500–600	1911	12	1612	629
≤500	1552	2	695	852
All	4833	148	4833	1684

a “All” includes all spectra shown, “Identified” includes spectra identified (blue) in the original FIREX library, “Reverse Score” gives distributions using the Reverse Identity scores, and “PFMD Removed” are numbers of query spectra with major perfluoromethyldecalin peaks removed.

Of the original 148 identifications made using NIST 2014, 108 were reidentified using NIST 2023, 32 IDs were changed, and 9 could not be reidentified. Two of the latter compounds (three spectra) were not in the NIST library, two may have been mislabeled, and 4 appeared to generate scores too low to be reported. These identifications were manually confirmed with the NISTMS user interface program. It is noteworthy that nearly a third of the identifications were made for spectra having scores below 700, well below the typical minimal acceptance threshold of 800. The following discussion shows that the score alone is not always a sufficient criterion for identification.

### Identification Probability

**Figure 2:**
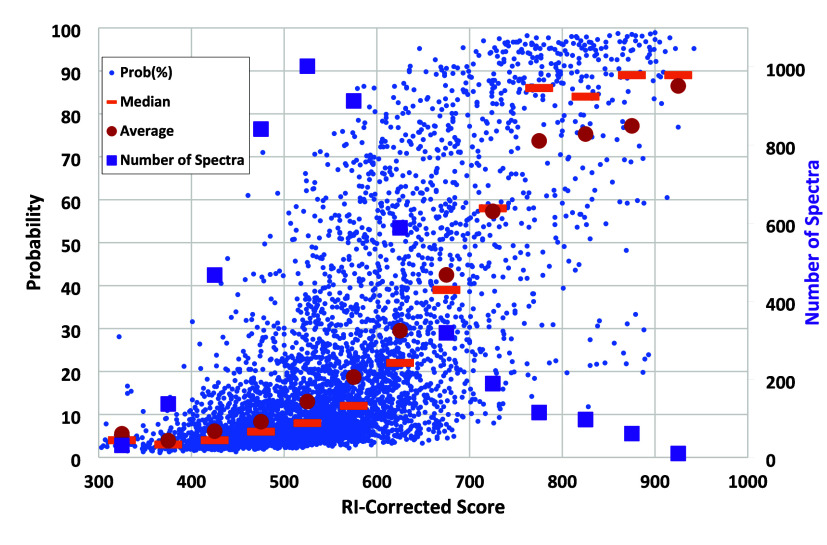
Distribution of reported “Probabilities” as a function of retention-index corrected Identity scores for FIREX data, with medians computed for bins of 50 score units shown as orange bars, averages as red circles, and numbers of spectra (right axis) as purple squares.

The uniqueness of an identification relative to other library spectra is reported as its “Probability”,^2^ assuming the matching compound is represented in the library. Identification probabilities for all spectra in the FIREX data set are shown in Figure 2 as a function of RI-corrected Identity scores. For the most confident identifications (above 800), an examination of low probability values finds that they arise from classes of isomers with similar spectra and retention times, such as aromatic and double bond positional isomers and TMS derivatized stereoisomeric sugars. At lower scores, due to an increasing number of false identifications, average score “densities” at the top of hit lists are much higher and computed “Probabilities” far lower.

The relatively stable level of median Probability of 75% at higher scores in Figure 2 suggests that this value is characteristic of a good identification for unique spectra. However, such high values are also found for a small proportion of lower scoring identifications, as observed in the upper left half of Figure 2. Individual examination confirmed that Probability values greater than 75% even at low score noticeably increased their identification confidence. We therefore suggest the following expression as a score correction, *Corr*, for Probability values above 75%:
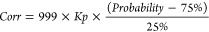
Through trial and error, a weighting factor of *Kp* = 0.25 was found as effective for this data set and is used in later illustrations. Using eq 1, this value leads to an increase of IDs with corrected scores above 800 of 64%. The central idea here is that spectrum uniqueness contributes to its identifiability by a library search and that if a match factor is intended to correlate with identification confidence, it should be increased with increasing spectrum uniqueness. However, this equation is intended only to illustrate the idea, with both the functional form and weighting factor subject to future refinement.

As evident in Figure 2, high Probabilities can be found at scores well below 750. Inspection of specific cases clearly showed that they were more likely to be correct than low Probability identifications at the same score. An example is spectrum for compound labeled UNK_4283 which has an initial score of 738, but with a computed Probability of 96% due to the next highest score of 580, the corrected score is raised to 855. Another is reported as “Benzoic Acid, TMS stereoisomer 1” in the FIREX collection with a score of 801 and a Probability of 91%, leading to a corrected score of 909. Finally, UNK_2852 identifies 1-heptadecanol with an initial score of 697, but with a Probability of 91% the score rises to 801. These cases are shown in the Supplemental Figure S3.

In cases where high scoring isomers at the top of a hit list have similar Probabilities, a large gap in scores can separate them from lower rank members. Such cases comprise the bulk of the high score, low probability identifications in Figure 2. In these cases, the probabilities of the isomer groups may be summed to yield a net probability for the group. These groups may be taken as those above the largest score difference in the hit list. An example are two aromatic positional isomers found for spectrum UNK_1951 (top hit is methyl homovanillate, TMS, the second isomer) at scores of 851 and 834 with the third ranked hit having a score of 624, elevating the probability from 64% to 99%. In such cases, a “group probability” is taken as the sum of the individual Probabilities. This value is given as an output in the library described later.

### Compound Ubiquity

A critical step in any nontargeted identification is the confirmation of the plausibility that the compound might actually be found in the sample under study. In fact, this assessment of “prior probability” is often the key missing component of any computer-based library search identification method^14^ and is expected to be provided by the analyst. In multicomponent targeted studies, this problem can be dealt with by searching a library containing only compounds expected to be present in the sample, although even here relative likelihoods are generally not provided. For nontargeted studies, the subject of this work, we propose and test the Compound Ubiquity Index, which is, in effect, a citation index that reports the numbers of 58 diverse chemical collections that reference each compound in the library (Supplementary Table 1). This is intended to represent the general likelihood that a compound is present in a naturally occurring mixture.

**Figure 3:**
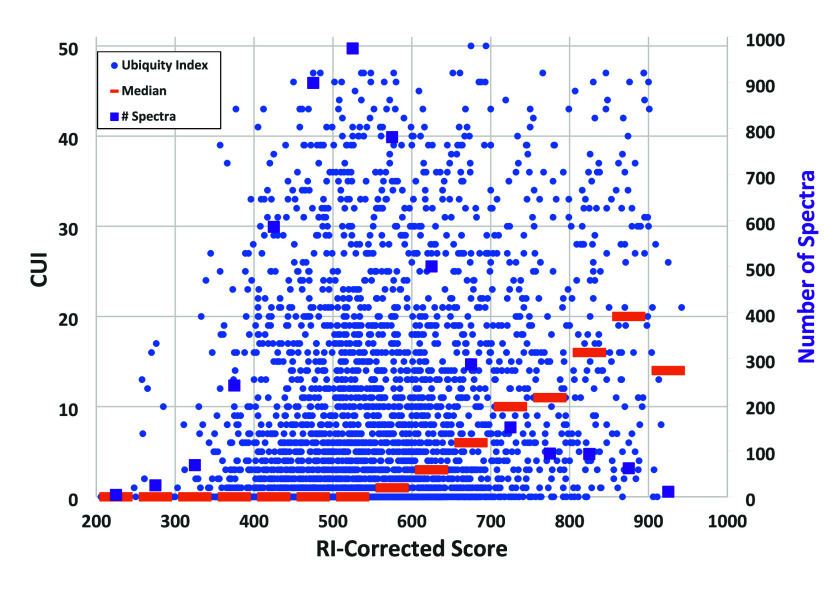
Compound Uniquity Index (CUI) as a function of the RI-corrected Identity score, with median and average values (left axis) and numbers of spectra (right axis) using bins of 50 score units.

**Figure 4:**
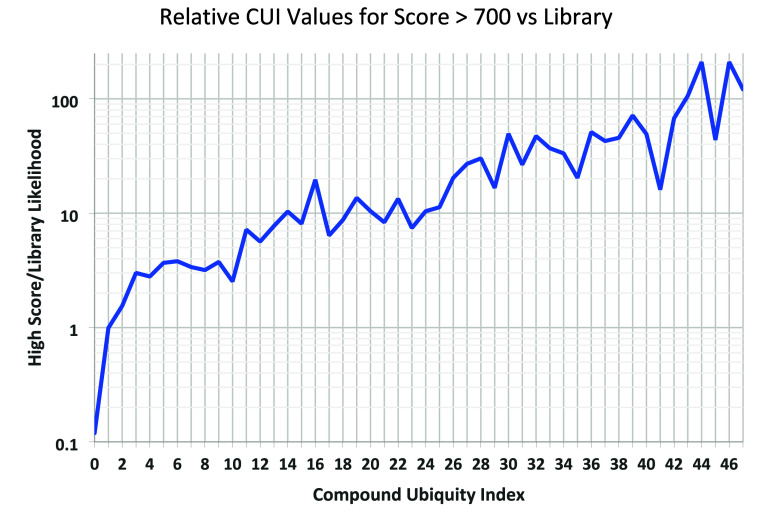
Log of CUI (output as OtherDBs in current NIST software) for 441 identifications with an RI-corrected Score >700 relative to all spectra in the NIST 2023 Library versus CUI.

Based on FIREX data, the dependence of CUI values on score is shown in Figure 3, along with median and average values and numbers of spectra. Figure 4 shows the increasing likelihood that a higher scoring ID has a high CUI relative to the likelihood of this CUI value occurring in spectra in the NIST 23 library with scores above 700. This curve changes little with increasing score cutoff. For the data set under study, a CUI of 0 reduces the inherent likelihood of identification relative to a random compound in the library by nearly a factor of 10, while a value of 20 increases this likelihood by a factor of 10.

The linearity of log (CUI) above CUI = 1 in Figure 4 suggests a simple score adjustment for the present data of
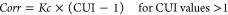
with a score penalty of −10 × *Kc* for CUI = 0 and no correction for CUI = 1, where *Kc* is an empirical weighting factor. Based on an examination of relevant results, we suggest a value of *Kc* = 2, which leads to an increase in numbers of IDs with a score of 800 or higher by 27%. This correction is consistent with the finding that an absolute difference in score correlates with the relative likelihood of being correct.^2^ The optimal value for *Kc* may depend on the nature of the sample. As for “probability” corrections described earlier, the purpose of this correction is to adjust confidence expressed by the match factor according to the CUI of an individual compound as indicated in Figure 4.

One may roughly compare these CUI score corrections to values currently used for Probability calculations,^2^ which are also derived from analysis of correct identifications based on hit list scores. From Figure 4, a 2-fold change in CUI occurs near a CUI of 2.5, leading to *Corr* of 5, while the currently used Probability correction for a 2-fold change suggests a change of 28 score units. Hence, by this measure, the proposed CUI weight is quite conservative.

A case in which this correction reorders the hit list is UNK_1978. In this case, the best scoring hit is (*E*)-5-octadecene with a score of 887 and *dRI* of 7, but a CUI of only 3. On the other hand, the fourth hit, 1-octadecene, has a score lower by 22 with a *dRI* of 2, but a much higher CUI of 26 (reported by NIST software as “OtherDB”). Using the score adjustment, the relative score is raised by 26 – 3 × 2 = 46, leading to a score of 901, placing it at the top of the hit list by 10 units. Another illustration of the application of CUI scoring is for the top scoring identification of 1,3-dimethoxy-2-hydroxybenzene TMS with a relatively low CUI of 5 and score of 769 and *dRI* of 13, while the second highest scoring identification is syringol TMS with the much higher CUI of 26 and even better *dRI* of 7. Application of the above score correction method elevates the score of the more common compound syringol by 50 score units to become the top hit at 797.

### Reverse Identity Search and Spectrum Contamination

The presence of peaks in a spectrum from coeluting compounds or background ions reduces identification confidence. This is of particular concern for compounds of low abundance since as their concentrations decrease, the relative intensities of contaminant and noise peaks increase. Moreover, the number of potentially interfering compounds in complex mixtures increases with decreasing concentration. Such spurious peaks are evident in many spectra shown in the Supporting Information. Contaminant peaks, especially at high masses, often above the molecular ion, can significantly reduce scores to well below a fixed identification limit. To reduce the severity of that problem, the Reverse Identity Search score was combined with the Identity Search score. Note that a confident match of a query spectrum requires that it contains all major peaks in the library spectrum. Nonmatching contaminant peaks are discarded by the Reverse Search. For the present data set, a combined score is taken as the average of the RI-corrected Identity and Reverse scores. This led to a net increase in scores at or above 800 of 21%. This is illustrated in Supplemental Figure S6, where the spectrum identified as phenanthrene in the FIREX library gave an Identity score of 682, but a Reverse score of 861. This correction elevates the score by 90 and with a CUI of 29 leads to a corrected score of 822. Ultimately, anthracene has a closer RI (*dRI* of 5 vs 15) and a slightly higher corrected score, so it would be predicted to be the more likely identification.

UNK_3667 provides another example shown in Figure S6, where high mass contaminants led to an Identity Search score of 696 and a Reverse score of 912, for 4-methylphenanthrene, boosting the score by 108 to a corrected score of 804. However, it is difficult to distinguish such isomers as reflected by the low Probability of 31%, hence the exact structure cannot be confidently determined. If noise peaks were not a problem due to careful deconvolution of query spectra, then this correction need not be used.

### Hybrid Similarity Search

**Figure 5:**
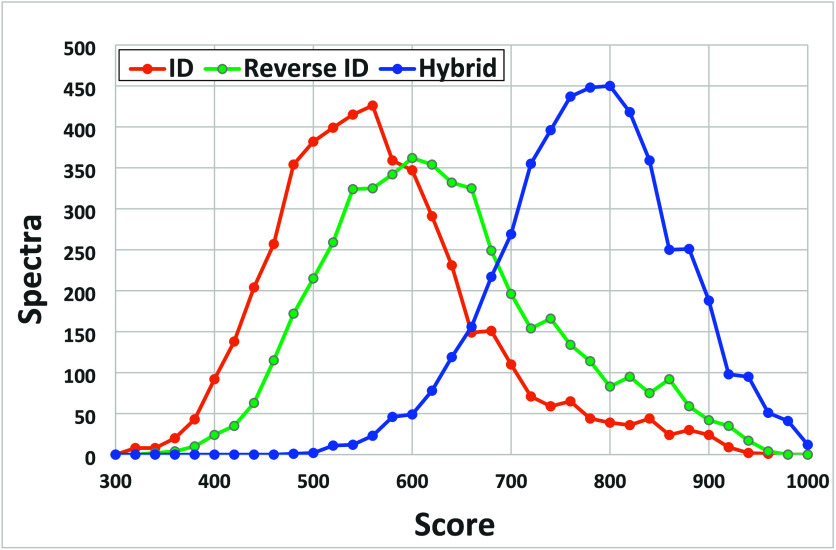
Top Hit Score Distribution for RI-corrected Identity (orange), Reverse ID (green), and Hybrid Similarity (blue) scores (using 20 score unit bins).

To examine the ability of HSS to assist in the elucidation of unidentified FIREX spectra, coarse molecular mass (M) estimates were added to the spectra prior to running through MSPepsearch to enable HSS analysis. For spectra containing the characteristic TMS 73 Da peak, molecular mass estimates were made by first attempting to find a pair of significant [M]^+^ and [M-15]^+^ Da peaks (the latter above 5% relative abundance). When this pair could not be found and for all other spectra, the molecular mass was simply taken as the highest nonisotopic mass with abundance above 5%. Figure 5 compares the resulting score distribution with the RI-corrected Identity and Reverse scores described earlier. The HSS scores are often far higher since the score increases when neutral loss peak matching can lead to higher scores and potential retention index penalties are not applied. Consequently, hit lists are often far more crowded with high scoring hits and require further analysis. Examples are presented later and in Supplemental Figure S1.

The hybrid search can help to identify compounds absent from the library when homologous compounds with related spectra are present in the library, and estimates of the molecular mass can be made. Since hits will generally differ in mass and retention index from the query spectrum, identification requires additional considerations: (1) DeltaMass must be converted to a plausible difference in chemical formula consistent with the matching library compound and a realistic chemical structure; (2) differences in observed and library retention indices (*dRI*) need to be within expectations; (3) hit lists often contain multiple high scoring hits (see Supplemental Figures S1 and S5), especially for compounds that are members of common classes, such as aliphatic alcohols or substituted aromatics; (4) shifted peaks must be consistent with fragmentation principles. Despite these requirements, in cases with high scoring HSS identifications having an easily interpreted DeltaMass value, such as a multiple of methylene (mass = 14 Da), high quality identifications can sometimes be readily made. Note that insertion of a methylene group generally corresponds to a change of about 100 Kovàts units^15^ and it is often possible to insert or delete methylene units to create plausible homologous structures. Moreover, multiple hits having similar structures may reinforce the identification. Finally, and importantly, the analyst must confirm the plausibility of the proposed structure in the mixture under investigation.

**Figure 6:**
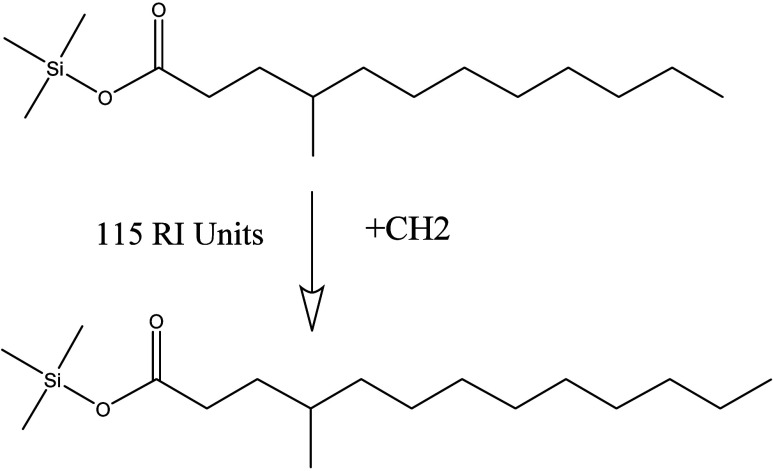
Plausible Hybrid Identification for UNK-2737 with a DeltaMass of 14 Da.

An example of an HSS aided identification is given in Figure 6 for the spectrum Unk_2737 in the original FIREX data set. The base 73 Da peak suggests that it is a TMS derivatized compound. Such compounds often contain a large [M-15]^+^ peak followed by a smaller molecular ion [M]^+^ with M as the molecular mass. In this case, it is 300 Da. In the hit list, 17 hits appear with a score over 900, all of them being TMS derivatives of linear or slightly branched alkyl acids (see Supplemental Figure S4). A promising hit is seventh in the hit list with a hybrid score of 915, a DeltaMass of 14 Da, and a library RI of 115 lower than the measured value of 1805, consistent with a single methylene difference, confirming the identification (Figure 6). The direct Identity Search score of the unshifted spectrum is only 688. Other hybrid identifications reinforce this identification.

**Figure 7:**
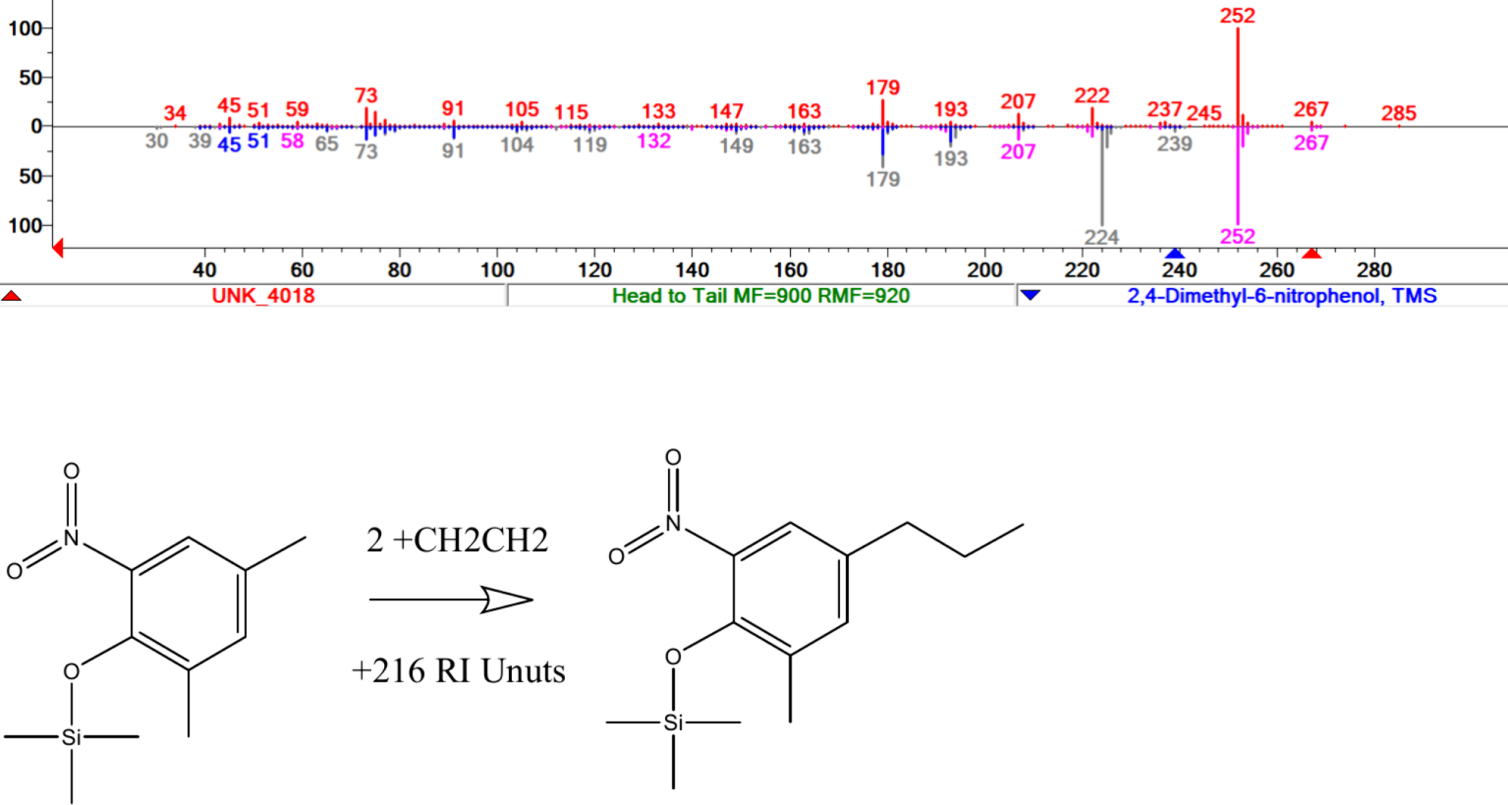
(top) Comparison of query (upper) and library spectra (lower) for a hybrid search, with gray library peaks shifted to pink positions by 28 Da. (bottom) Possible structure difference in library and query structures with 2 methylene groups added to the library structure. Insufficient information is available to establish the specific positional isomer.

Another HSS example is spectrum UNK_4018, which gives a very low direct score of 578 but using a molecular mass of 267 Da generates a top HSS hit of 900 for 2,4-dimethyl-6-nitrophenol with a DeltaMass of 28 Da (2 methylene units). This is consistent with the RI of the unknown being higher than that of the library compound by 216 retention units, which is close to a value expected for two methylene units. The comparison of spectra and plausible structure correction is shown in Figure 7.

A final example is UNK_1462 which was matched using HSS with a molecular mass of 300 Da, its most abundant high mass peak. The second rank match is 4-methylcatechol, 2-TMS with a HSS score of 942 and a DeltaMass of 12 Da. This corresponds to conversion of a methyl group to a vinyl group. The *dRI* is 125, nearly identical to the 129 RI difference in RI for the simplest aromatic methyl to vinyl conversion, namely toluene to styrene. Other hit list compounds support this assignment, as shown in Supplemental Figure S1a.

These illustrations show that the application of this method for such common chemical classes requires expertise and that specific isomeric structure can be uncertain, though perusal of high scoring hits and retention indices can find the specific chemical class and size. Two other illustrations of HSS identifications are presented in Supplemental Figure S5 (UNK_430 and UNK_1823).

The fact that the HSS does not employ RI penalties can aid identification in other ways. For example, spectrum UNK_1850 with a molecular mass of 354 Da yielded a 945 high scoring identification as the second rank in the hit list with DeltaMass = 0 (no peak shifting, so same score as for the Similarity search) for the library compound, trans-sinapyl alcohol diTMS. This compound was identified in another spectrum, but the expected RI was higher by 120 units. Examination of data from our lab revealed the cis isomer to elute 118 RI units earlier than the trans isomer, confirming that to be the actual identification. Of course, one might have arrived at the same conclusion by turning RI-penalization off, but HSS provides the same information while trying to identify compounds not in the library.

### Unidentified Spectra

**Figure 8:**
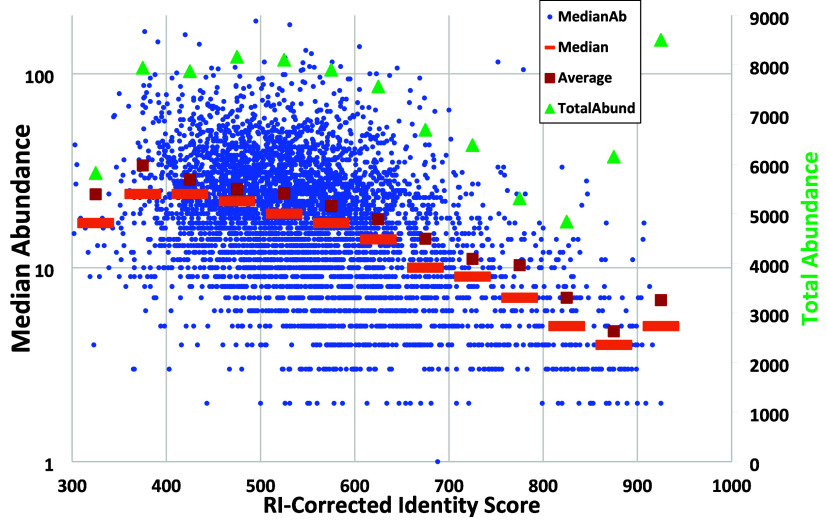
Distribution of median spectrum abundances vs RI-corrected Identity scores (maximum 999). Median and average values are shown in orange and red, respectively, using bins of 50 score units with abundance units (right axis) 10 times the percentage of base peak. Sums of peak intensities are shown in green and on the right axis. Median values are calculated prior to PFMD removal.

**Figure 9:**
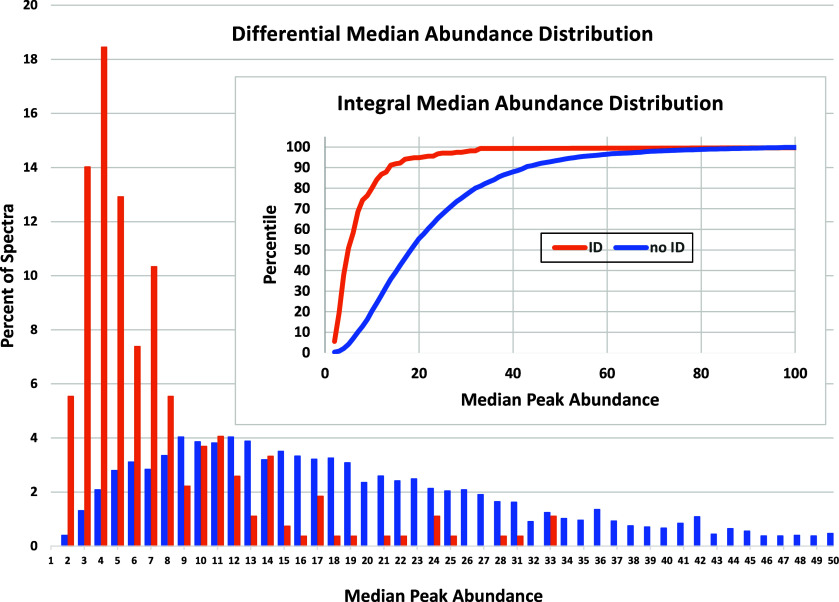
Distributions of the median abundance of high scoring (ID) and low scoring (noID) spectra. About 78% of identified spectra have median abundances of 10 or less while only 24% of unidentified spectra fall in this range. The main plot is a “differential” plot of spectra at each abundance (10 times percent) and the inset is an integral plot showing this in an integral form (number of spectra having less than or equal to *x*-axis median abundance).

Of the 4833 spectra in the data set, approximately 90% of them could not be identified, even with a score threshold of 700. To explore the reasons for this low level of identification, several features of the unidentified spectra were compared to those of the identified spectra. Most strikingly, identified high scoring spectra had distinctly lower median peak abundances than did low scoring unidentified spectra. Figures 8 and 9 show that median abundance values varied from 0.49% for high scoring, mostly identifiable spectra to 1.73% of the base peak for low scoring, unidentifiable spectra. Numbers of peaks and sums of base-peak normalized abundances increased, though varied by less than a factor of 2. This is consistent with the bulk of unidentified spectra having more contaminant peaks and weaker signals. Note that a spectrum consisting of mostly noise peaks will often have a high sum of reported abundances in a base peak normalized spectrum. Examination of lower scoring identifications showed that seemingly random contaminant peaks, often extending to high masses, were a principal reason for the low scores. Examples are shown in Supplemental Figure S2.

Occasionally, good identifications can have high median abundances. Two cases were found with median values higher than 10% of the base peak. Both matching library spectra had over 100 peaks, an unusually high number. They were UNK_4305 with score 752, median abundance = 12% and UNK_4534 with score 779 and median abundance of 11%. The next lowest median value was 33 or 3.3%! Both are shown in Supplemental Figure S5.

For the FIREX data set, as shown in Figure 9, a low median peak abundance is correlated with high score, hence spectrum identifiability. While the dynamic range of peak abundances for a high-quality spectrum is generally high due to the presence of minor fragment ions and their isotopes, a spectrum with a relatively high median abundance spectrum can indicate that the smaller peaks are near the detection limit and hence more likely to be minor contaminating ions. Since such peaks have low absolute but high relative abundances, their summed abundances will often be higher than summed abundances in high quality spectra.

### Composite Scoring and Annotation

A corrected score may be derived using eq 1, with *MF1* as the combined Identity and Reverse score, Corr as the sum of the Probability and CUI corrections, and *dMF* is 999 – *MF1* for positive *Corr* and *MF1* for negative *Corr*. This new score increases the number of IDs above a score of 800 relative to the RI-corrected Identity score by a factor of 2.2. The application and benefit of a composite score is illustrated by the reported identification of UNK_4684 as decanoic acid, TMS. Without correction, this spectrum produces the very low score of 597 as shown as Supplemental Figure S7, even though it has closely matching RI, with *dRI* of 3 Kovàts units. However, based on eq 1, three factors serve to greatly increase its final score: (1) the Reverse Search score of 814 yields *MF1* = (597 + 814)/2 = 705.5 and *dMF* = 999 – 705.5 = 293.5; (2) the Probability of 92% yields a correction of 999 × 0.2(92 – 75)/25 = 135; (3) the CUI of 43 yields a correction of 2(43 – 1) = 84. Using eq 1, with *Corr* = 135 + 84 following calculation below yields a final, composite score of 830, indicative of a good identification.
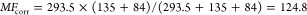

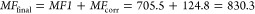


### Updated Data Set

A revised FIREX library in NIST format is available for download.^17^ It contains all spectra in the original FIREX data set with annotation added containing new scores and other information described in this paper. It can be accessed by publicly available NIST Search Software^12^ as well as any software distributed along with NIST EI libraries. Original names are replaced with names from the NIST 2023 library for scores over 750. Further details are provided in a readme.txt file provided with the data set.

## 4 Conclusion

This work describes the development and application of multiple factors derived from library searching that can increase the confidence in compound identification from their EI mass spectra. The FIREX GCxGC EI data set^6^ was employed as the source of data for developing and illustrating this analysis. It is shown how these factors can contribute significantly to the confirmation of compound identities and also enhance the selectivity of search algorithms.

Any identification of a nontargeted compound by GC-MS requires verification by an expert. The relative significance of specific peaks for specific identifications and the plausibility of a compound being detected in a sample are issues for a skilled analyst to resolve. The factors examined here are intended to aid this process. For this purpose, all needed information is derived from searches of the NIST 2023 EI library, which in addition to providing retention indices for all represented compounds, provides measures of compound ubiquity and spectrum uniqueness as well as a means of minimizing the effects of spurious spectral peaks. While consideration of individual factors may be used by analysts in identity validation, a simple method for combining these factors to derive an adjusted score is described. This enables analysts to assemble a list of compound identifications more quickly by making use of the additional selectivity provided by these factors in place of conventional measures of spectrum matching.

The factors examined for improving identification confidence were (1) the general ubiquity of each library compound, as derived from the Compound Ubiquity Index, the number of collections in which a compound has been cited, (2) reported “Probabilities” with each score, which provide a measure of the uniqueness of each query spectrum relative to the best matching library spectra, and (3) combining the Reverse Identity Score with the Identity Score, both RI-corrected, to reduce effects of contamination. Since each of these factors correlates with identification confidence, they can be used to modify a hitlist score based on confidence in correct identification rather than only on spectral similarity and retention index matching. An illustration of the use of these factors to derive a final score is given in the section on “Composite Score”. Using the present settings, corresponding percent increases of identified spectra over a score of 800 for these three corrections are (1) 27%, (2) 64%, and (3) 21%, respectively, leading to a net increase of a factor of 2.2. We emphasize that these methods are intended to be only illustrative and may be significantly refined later. Also described are applications of the Hybrid Similarity Search method for tentatively identifying compounds that are different, but similar to those that are represented in the library.^10^ Further, consideration of isotopic abundance ratios can be used for additional confirmation of identity.

In a comparison of identified with unidentified spectra, the latter were found to have significantly smaller ranges of peak abundance, as measured by their higher median abundances. Median values were 0.49% (0.49) for identified spectra and 1.73% for unidentified spectra. This and examination of low scoring matches showed that the cause of most spectra being unidentified in the FIREX data set was primarily a result of low signal strength and associated contamination. This simple factor may be of use to select unidentified spectra that are most likely to be identifiable by methods other than simple library searching.

## Data Availability

An updated FIREX mass spectral library in NIST library binary format and ASCII.txt format with.pdf documentation from ref (17) Microsoft Windows software for accessing this NIST library format is given in ref (11), accessible by any recent version of the NISTMS EI library software.

## Supporting Information Available

Sources used for the CUI (XLSX)
Supplemental figures (PDF)

## Abbreviations

*Corr*: Corrections to Match Factor (eq 1)
CUI: Compound Ubiquity Index
PFMD: Perfluoromethyldecalin
DeltaMass: Difference in molecular mass of query and matching library identification
*dMF*: Difference between match factors (score)
*dRI*: Difference in Kovàts retention indices between query and matching library identification
FIREX: Fire Influence on Regional to Global Environments Experiment
M: Molecular mass (Da)
*MF1*: Initial score (average of identity and reverse score)
*MFcorr*: total score adjustment
RI: Kovàts retention index
TMS: trimethylsilyl derivative
