# Supplementary material for: New Library-Based Methods for Nontargeted Compound Identification by GC-EI-MS
Source: J Am Soc Mass Spectrom. 2025 Jan 14;36(2):389–99. doi: 10.1021/jasms.4c00451 (PMC11844893; doi:10.1021/jasms.4c00451)
Supplement: Supplementary file 2 — js4c00451_si_002.pdf [file js4c00451_si_002.pdf]

Supporting Information

New library-based methods for non-targeted compound identification by GC-EI-MS

Deborah F. McGlynn[1], Lindsay D. Yee[2], H. Martin Garraffo[3], Lewis Y. Geer[3], Tytus D. Mak[3], Yuri A. Mirokhin[3], Dmitrii V. Tchekhovskoi[3], Coty N. Jen[2], Allen H. Goldstein[2], Anthony J. Kearsley[1], Stephen E. Stein[3*]

[1]Applied and Computational Mathematics Division, National Institute of Standards and Technology, Gaithersburg, MD 20899, USA
[2]Department of Environmental Science, Policy, & Management, University of California at Berkeley, Berkeley CA 94720
[3]Biomolecular Measurement Division, National Institute of Standards and Technology, Gaithersburg, Maryland 20899-8362, United States

* Corresponding author: stephen.stein@nist.gov

Supplemental Figure Section

Examples of library search results are presented here as screenshots of NISTMS v. 3.0 software searches using the NIST 2023 EI library. The FIREX query spectrum name is shown in the upper left section and its spectrum and text information on the upper right. The hit list of matching NIST spectra ordered by rank is at the lower left with the highlighted library spectrum and text shown at the lower right. Between the query spectrum (upper right) and library spectrum (lower right) is their comparison spectrum, shown here as a head-to-tail plot.

For all figures except 1 and 5, results are for RI-corrected identification analyses. Hit list columns (lower left) are, in the order: 1) Rank, 2) NIST Main or Replicate Library, 3) RI-corrected Identity Search Match Factor, 4) Identity Match Factor without RI penalty, 5) Reverse Score, 6) Probability, 7) Kovats Retention Index, 8) dRI or difference between Query and Library Kovats Retention Index, and in some figures additional columns are shown including 9) molecular mass and 10) otherDBs (CUI, Compound Ubiquity Index).

For Figures 1 and 5, Hybrid search results show additional fields, with columns 3) and 4) being the Hybrid score, 5) Original Similarity score (prior to Hybrid scoring), 6) Reverse Similarity score, 7) DeltaMass (difference in query and library molecular mass), 8) library retention index, and 9) dRI (difference between query and library RI)

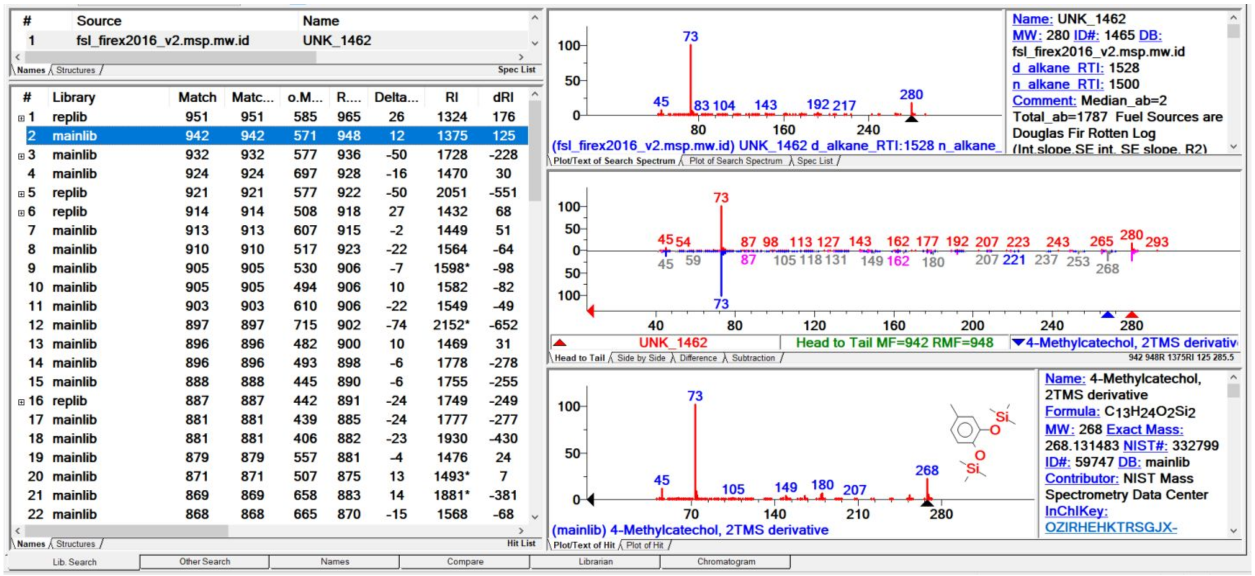

Source Name
1 fsl_firex2016_v2.msp.mw.id UNK_1462
Library Match Matc... o.M... R.... Delta... RI dRI
1 replib 951 951 585 965 26 1324 176
2 mainlib 942 942 571 948 12 1375 125
3 mainlib 932 932 577 936 -50 1728 -228
4 mainlib 924 924 697 928 -16 1470 30
5 replib 921 921 577 922 -50 2051 -551
6 replib 914 914 508 918 27 1432 68
7 mainlib 913 913 607 915 -2 1449 51
8 mainlib 910 910 517 923 -22 1564 -64
9 mainlib 905 905 530 906 -7 1598* -98
10 mainlib 905 905 494 906 10 1582 -82
11 mainlib 903 903 610 906 -22 1549 -49
12 mainlib 897 897 715 902 -74 2152* -652
13 mainlib 896 896 482 900 10 1469 31
14 mainlib 896 896 493 898 -6 1778 -278
15 mainlib 888 888 445 890 -6 1755 -255
16 replib 887 887 442 891 -24 1749 -249
17 mainlib 881 881 439 885 -24 1777 -277
18 mainlib 881 881 406 882 -23 1930 -430
19 mainlib 879 879 557 881 -4 1476 24
20 mainlib 871 871 507 875 13 1493* 7
21 mainlib 869 869 658 883 14 1881* -381
22 mainlib 868 868 665 870 -15 1568 -68
(fsl_firex2016_v2.msp.mw.id) UNK_1462 d_alkane_RTI:1528 n_alkane_
Name: UNK_1462
MW: 280 ID#: 1465 DB: fsl_firex2016_v2.msp.mw.id
d_alkane_RTI: 1528
n_alkane_RTI: 1500
Comment: Median_ab=2 Total_ab=1787 Fuel Sources are Douglas Fir Rotten Log
UNK_1462
Head to Tail MF=942 RMF=948
4-Methylcatechol, 2TMS derivativ
(mainlib) 4-Methylcatechol, 2TMS derivative
Name: 4-Methylcatechol, 2TMS derivative
Formula: C13H24O2Si2
MW: 268 Exact Mass: 268.131483 NIST#: 332799
ID#: 59747 DB: mainlib
Contributor: NIST Mass Spectrometry Data Center
InChIKey: OZIRHEHKTRSGJX-
Lib. Search
Other Search
Names
Compare
Librarian
Chromatogram

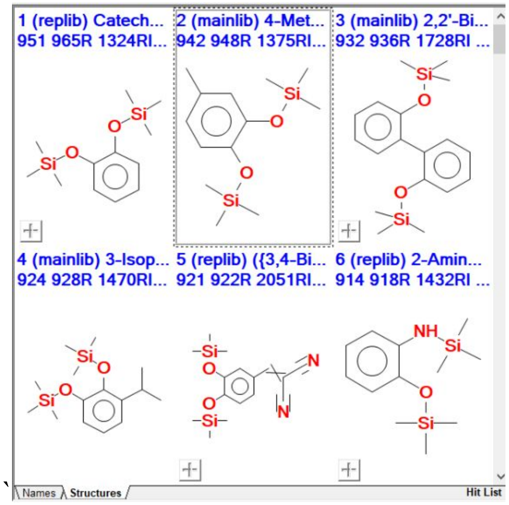

1 (replib) Catech... 951 965R 1324RI...
2 (mainlib) 4-Met... 942 948R 1375RI...
3 (mainlib) 2,2'-Bi... 932 936R 1728RI ...
4 (mainlib) 3-Isop... 924 928R 1470RI...
5 (replib) ({3,4-Bi... 921 922R 2051RI...
6 (replib) 2-Amin... 914 918R 1432RI ...
Names Structures
Hit List

Figure S1a.

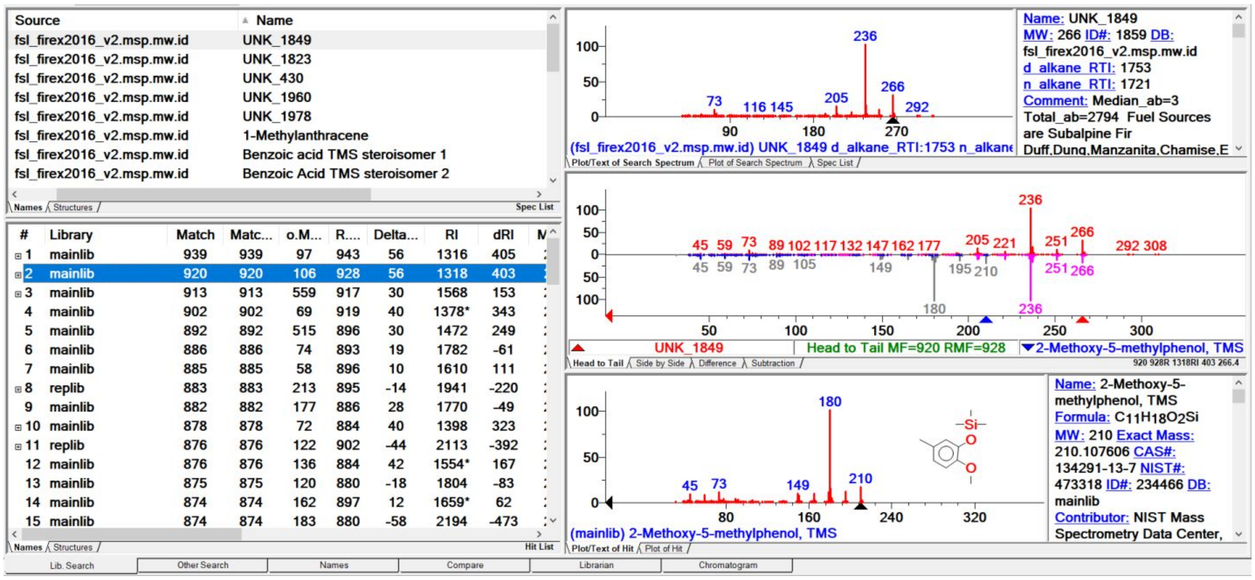

Figure S1b.

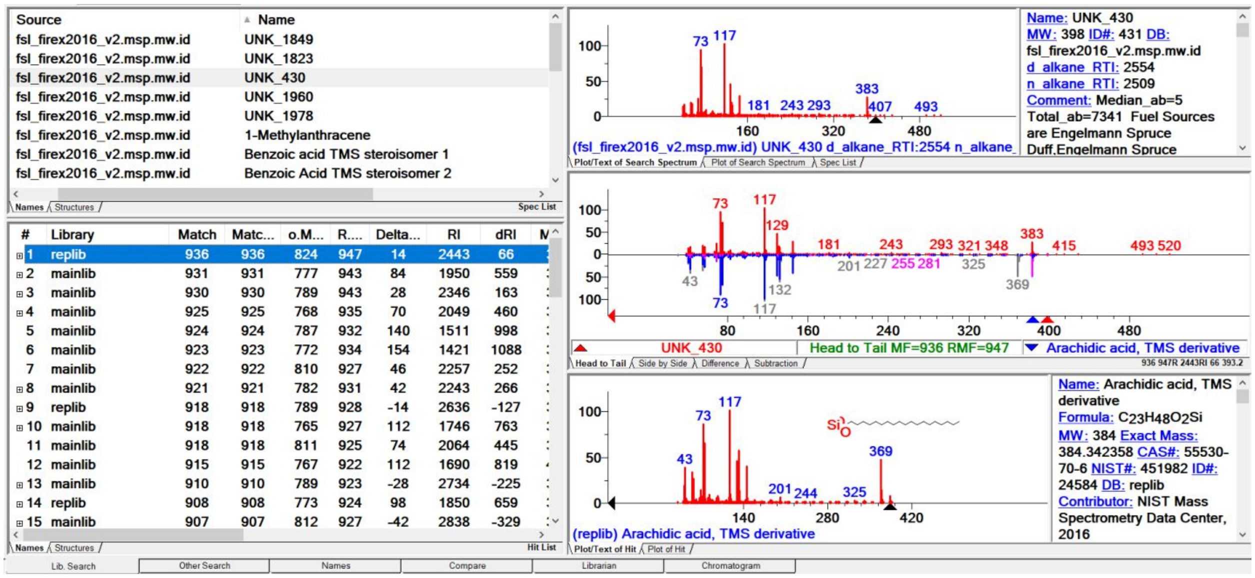

Figure S1c.

Three examples, S1a-c of Hybrid Search results showing the many high scores that can appear for this search type. Column meanings given at top of this section. S1a: DeltaMass = 12, corresponds to methyl conversion to a double bond styrene-like structure, with dRI consistent with the toluene to styrene transformation; S1b: DeltaMass = 56 = 4 x 14 = 4 CH2 with dRI=405, consistent with insertion of an n-butyl group into the chain of Arachidic acid; S1c: Suggests inserted methylene (DeltaMass = 14 Da), but less than 100 RI difference consistent with branching.

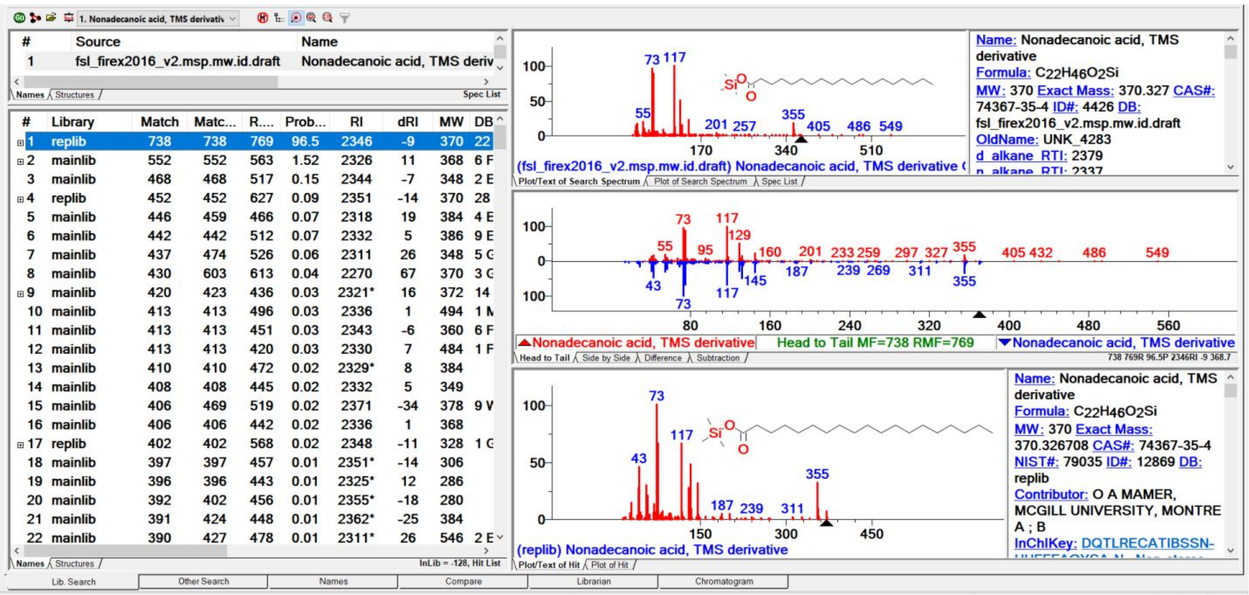
1. Nonadecanoic acid, TMS derivativ
Source Name
1 fsl_firex2016_v2.msp.mw.id.draft Nonadecanoic acid, TMS deriv
Library Match Matc... R.... Prob... RI dRI MW DB
1 replib 738 738 769 96.5 2346 -9 370 22
2 mainlib 552 552 563 1.52 2326 11 368 6 F
3 mainlib 468 468 517 0.15 2344 -7 348 2 E
4 replib 452 452 627 0.09 2351 -14 370 28
5 mainlib 446 459 466 0.07 2318 19 384 4 E
6 mainlib 442 442 512 0.07 2332 5 386 9 E
7 mainlib 437 474 526 0.06 2311 26 348 5 G
8 mainlib 430 603 613 0.04 2270 67 370 3 G
9 mainlib 420 423 436 0.03 2321* 16 372 14
10 mainlib 413 413 496 0.03 2336 1 494 1 M
11 mainlib 413 413 451 0.03 2343 -6 360 6 F
12 mainlib 413 413 420 0.03 2330 7 484 1 F
13 mainlib 410 410 472 0.02 2329* 8 384
14 mainlib 408 408 445 0.02 2332 5 349
15 mainlib 406 469 519 0.02 2371 -34 378 9 V
16 mainlib 406 406 442 0.02 2336 1 368
17 replib 402 402 568 0.02 2348 -11 328 1 G
18 mainlib 397 397 457 0.01 2351* -14 306
19 mainlib 396 396 443 0.01 2325* 12 286
20 mainlib 392 402 456 0.01 2355* -18 280
21 mainlib 391 424 448 0.01 2362* -25 384
22 mainlib 390 427 478 0.01 2311* 26 546 2 E
InLib = -128, Hit List
(fsl_firex2016_v2.msp.mw.id.draft) Nonadecanoic acid, TMS derivative
Name: Nonadecanoic acid, TMS derivative
Formula: C22H46O2Si
MW: 370 Exact Mass: 370.327 CAS#: 74367-35-4 ID#: 4426 DB: fsl_firex2016_v2.msp.mw.id.draft
OldName: UNK_4283
d_alkane_RTI: 2379
Nonadecanoic acid, TMS derivative Head to Tail MF=738 RMF=769 Nonadecanoic acid, TMS derivative
738 769R 96.5P 2346RI -9 368.7
(replib) Nonadecanoic acid, TMS derivative
Name: Nonadecanoic acid, TMS derivative
Formula: C22H46O2Si
MW: 370 Exact Mass: 370.326708 CAS#: 74367-35-4
NIST#: 79035 ID#: 12869 DB: replib
Contributor: O A MAMER, MCGILL UNIVERSITY, MONTRE A ; B
InChIKey: DQTLRECATIBSSN-
Lib. Search
Other Search
Names
Compare
Librarian
Chromatogram

S2a.

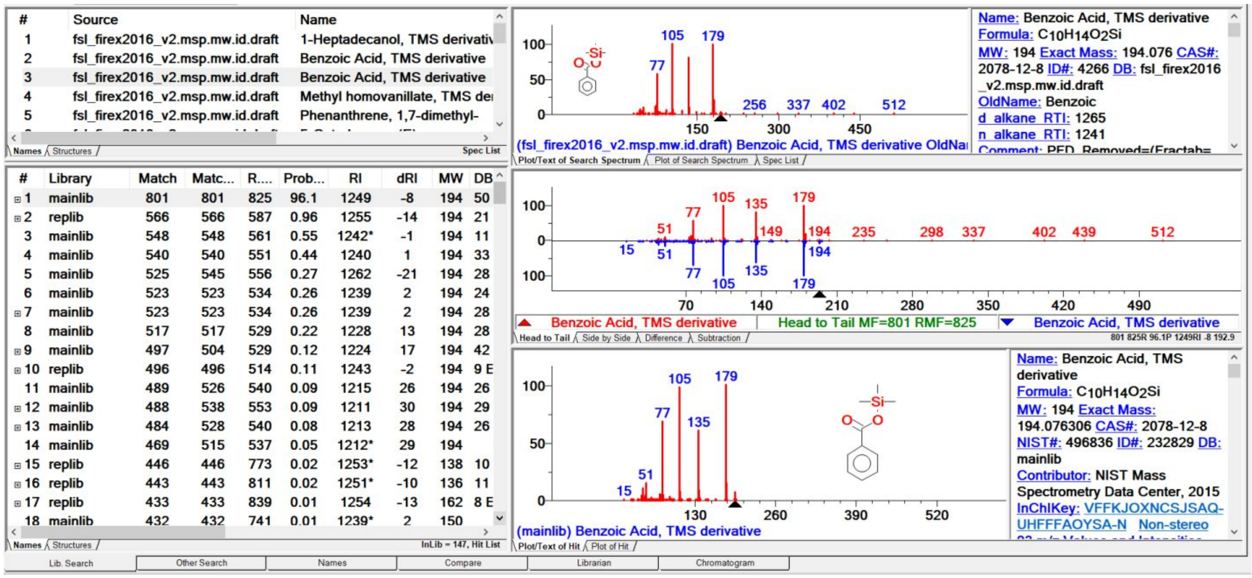
Source Name
1 fsl_firex2016_v2.msp.mw.id.draft 1-Heptadecanol, TMS derivativ
2 fsl_firex2016_v2.msp.mw.id.draft Benzoic Acid, TMS derivative
3 fsl_firex2016_v2.msp.mw.id.draft Benzoic Acid, TMS derivative
4 fsl_firex2016_v2.msp.mw.id.draft Methyl homovanillate, TMS de
5 fsl_firex2016_v2.msp.mw.id.draft Phenanthrene, 1,7-dimethyl-
Library Match Matc... R.... Prob... RI dRI MW DB
1 mainlib 801 801 825 96.1 1249 -8 194 50
2 replib 566 566 587 0.96 1255 -14 194 21
3 mainlib 548 548 561 0.55 1242* -1 194 11
4 mainlib 540 540 551 0.44 1240 1 194 33
5 mainlib 525 545 556 0.27 1262 -21 194 28
6 mainlib 523 523 534 0.26 1239 2 194 24
7 mainlib 523 523 534 0.26 1239 2 194 28
8 mainlib 517 517 529 0.22 1228 13 194 28
9 mainlib 497 504 529 0.12 1224 17 194 42
10 replib 496 496 514 0.11 1243 -2 194 9 E
11 mainlib 489 526 540 0.09 1215 26 194 26
12 mainlib 488 538 553 0.09 1211 30 194 29
13 mainlib 484 528 540 0.08 1213 28 194 26
14 mainlib 469 515 537 0.05 1212* 29 194
15 replib 446 446 773 0.02 1253* -12 138 10
16 replib 443 443 811 0.02 1251* -10 136 11
17 replib 433 433 839 0.01 1254 -13 162 8 E
18 mainlib 432 432 741 0.01 1239* 2 150
InLib = 147, Hit List
(fsl_firex2016_v2.msp.mw.id.draft) Benzoic Acid, TMS derivative OldNa
Name: Benzoic Acid, TMS derivative
Formula: C10H14O2Si
MW: 194 Exact Mass: 194.076 CAS#: 2078-12-8 ID#: 4266 DB: fsl_firex2016_v2.msp.mw.id.draft
OldName: Benzoic
d_alkane_RTI: 1265
n_alkane_RTI: 1241
Benzoic Acid, TMS derivative Head to Tail MF=801 RMF=825 Benzoic Acid, TMS derivative
801 825R 96.1P 1249RI -8 192.9
(mainlib) Benzoic Acid, TMS derivative
Name: Benzoic Acid, TMS derivative
Formula: C10H14O2Si
MW: 194 Exact Mass: 194.076306 CAS#: 2078-12-8
NIST#: 496836 ID#: 232829 DB: mainlib
Contributor: NIST Mass Spectrometry Data Center, 2015
InChIKey: VFFKJOXNCSJSAQ-UHFFFAOYSA-N Non-stereo
Lib. Search
Other Search
Names
Compare
Librarian
Chromatogram

S2b .

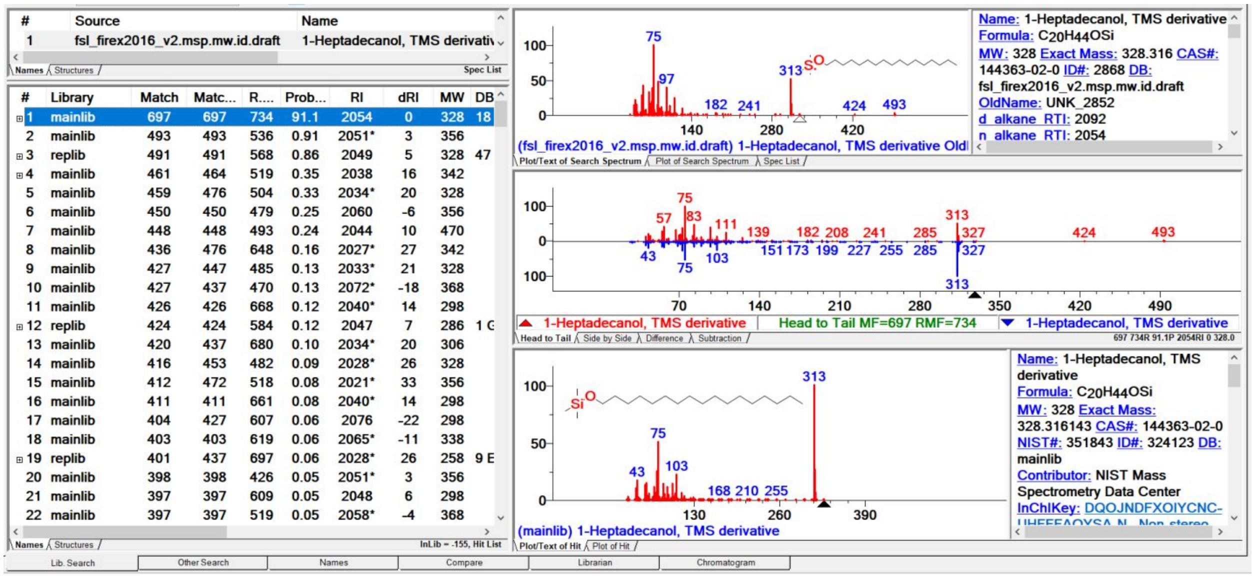

S2c

Figure S2a-c. Three RI-corrected Identity search identifications assisted by their high ‘Probabilities’. (1) S2a: UNK_4283 which has a RT-corrected Identity Score of 721 and RI within 9 units of the reference value, but with a Probability of 96% due to next highest score of 580. With the correction described in the text this score rises to 833. (2) S2b: Reported as Benzoic Acid, TMS derivative in the FIREX collection but with a score of only 694 with a low RI deviation of 8 units and a Probability of 96%, leading to a corrected score of 802. (3) S2c. Initial spectrum UNK_2852 with a 91% Probability identifies 1-heptadecanol, TMS leading to a corrected score of 801 using only this factor. Results involved non-indexed search to produce most reliable Probabilities.

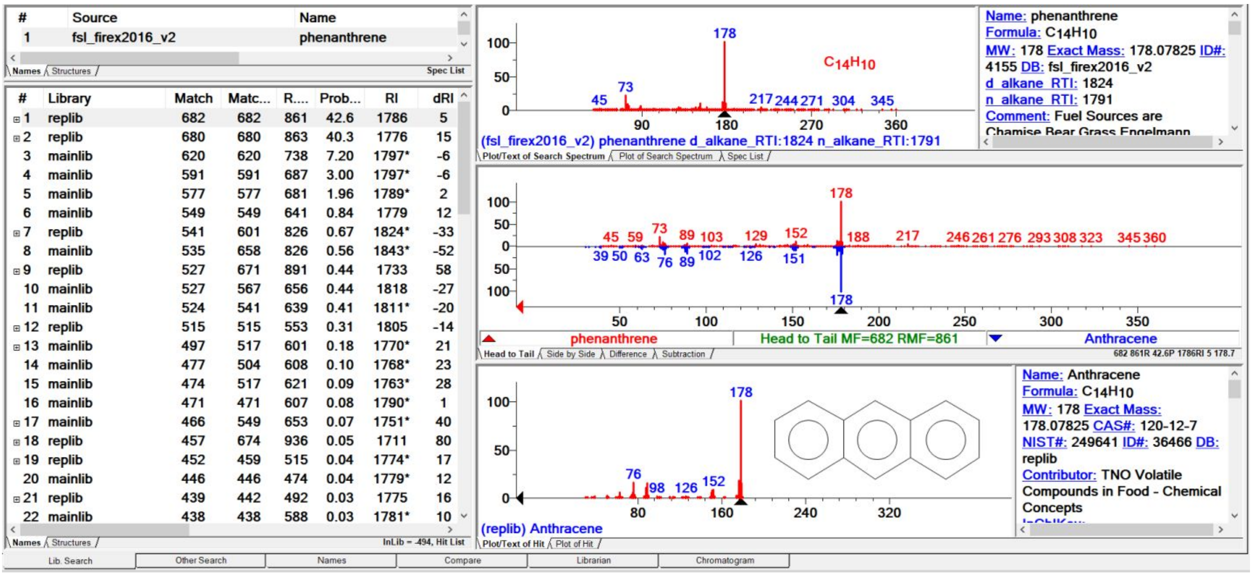

Figure S3a.

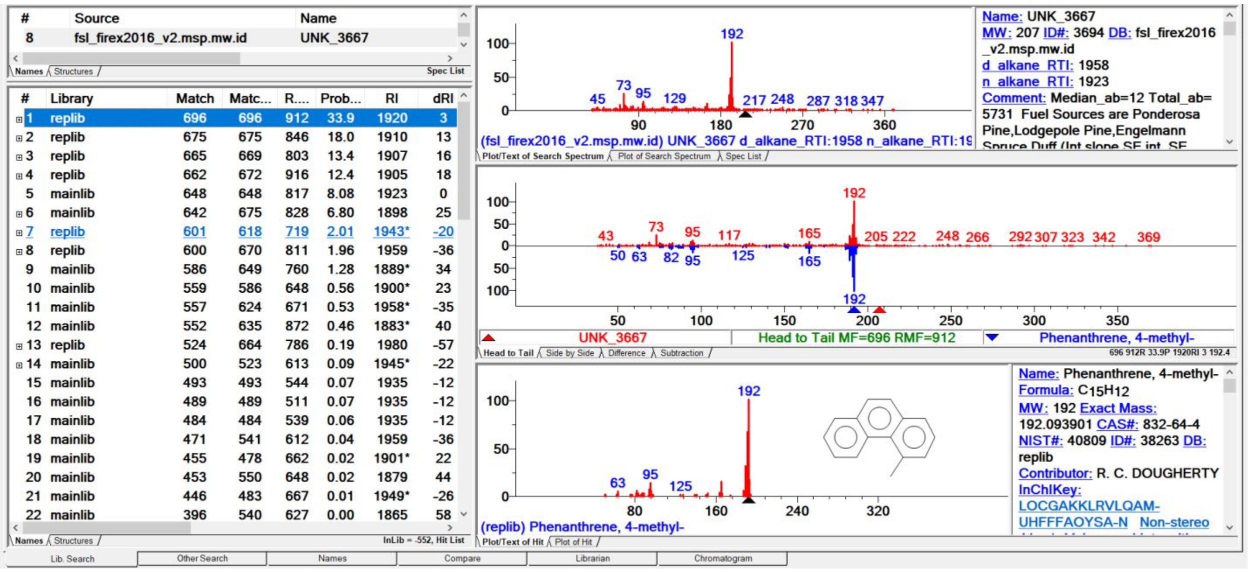

Figure S3b.

Figure 3a,b. Examples of two RI-corrected Identity searches of spectra whose Reverse Search scores are much higher than from the Identity Search due primarily to rejection of non-matching high mass peaks in the query spectra.

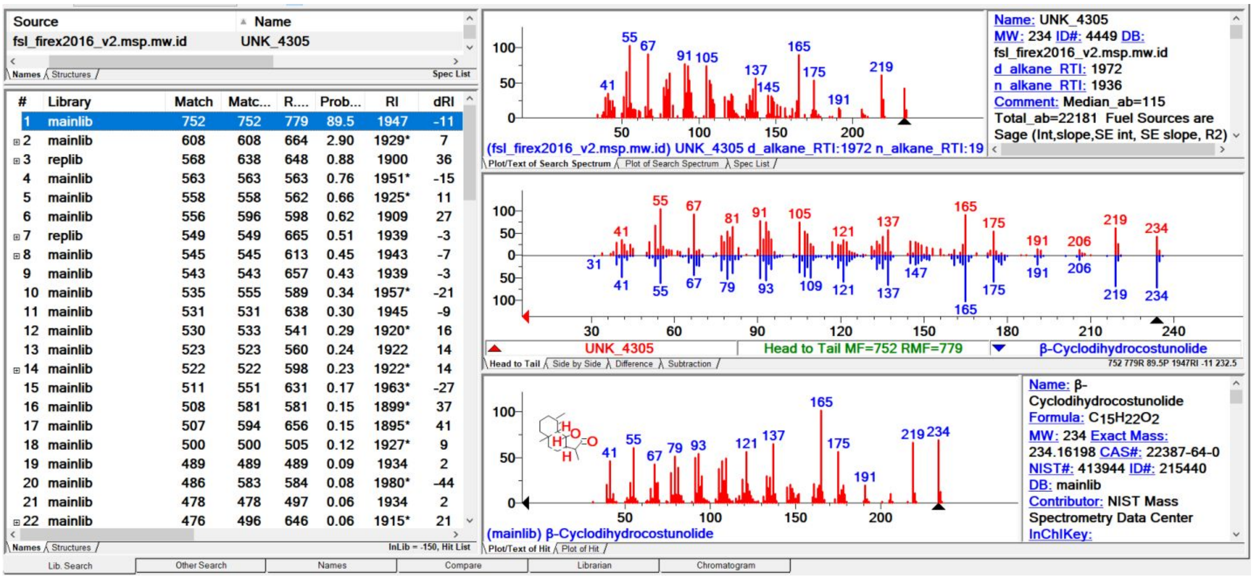

S4a.

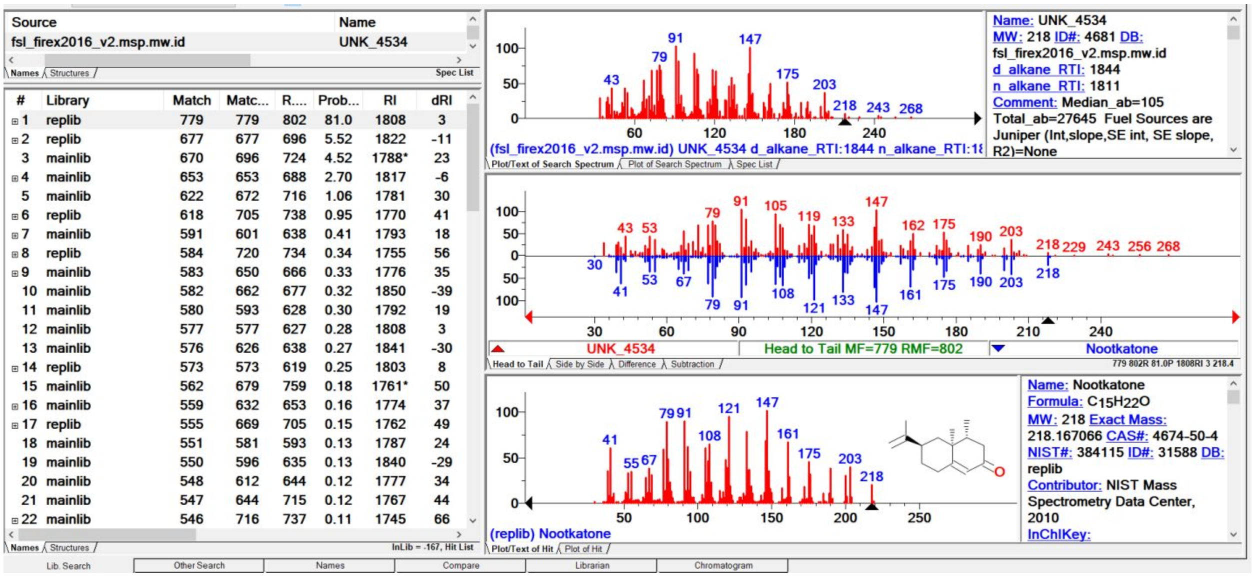

S4b.

Figure S4a,b. Two RI-corrected Identity search identifications with unusually high median abundances. They are S4a UNK_4305 with score 752, median abundance = 12% and S4b UNK_4534 with score 779 and median abundance of 105. The next lowest median abundance value of any identification in the FIREX dataset was 33 (Figure 7).

#	Library	Match	Matc...	o.M...	R....	Delta...	RI	dRI	MW(RI)	MW	DBs	Formula	Name
1	mainlib	938	938	767	957	56	1421	384	297.8	244	16 E...	C13H28O2Si	8-Methylnonanoic acid, trimethylsilyl ester
⊞ 2	mainlib	931	931	758	955	14	1746	59	294.3	286	32 W...	C16H34O2Si	Tridecanoic acid, TMS derivative
3	mainlib	928	928	798	932	-24	2064	-259	287.7	324		C19H36O2Si	Palmitic acid alkyne, TMS
4	mainlib	927	927	746	945	70	1322	483	297.6	230	14 C...	C12H26O2Si	7-Methyloctanoic acid, TMS
5	mainlib	923	923	746	940	70	1331	474	296.4	230	4 GM	C12H26O2Si	6-Methyloctanoic acid, TMS
6	mainlib	917	917	764	935	42	1511	294	299.2	258	2 GM	C14H30O2Si	7-Methyldecanoic acid, TMS
7	mainlib	915	915	739	936	14	1690	115	302.1	286	2 GM	C16H34O2Si	4-Methyldodecanoic acid, TMS
8	mainlib	914	914	795	917	-52	2257	-452	288.7	352	6 EGM	C21H40O2Si	17-Octadecynoic acid, TMS derivative
9	mainlib	912	912	738	927	30	1644	161	292.5	270	9 EF...	C15H30O2Si	11-Dodecenoic acid, TMS
⊞ 10	mainlib	911	911	708	932	56	1450	355	293.7	244	43 W...	C13H28O2Si	Decanoic acid, TMS derivative
⊞ 11	mainlib	910	910	768	934	-14	1950	-145	293.7	314	29 W...	C18H38O2Si	Pentadecanoic acid, TMS derivative
⊞ 12	replib	909	909	732	933	28	1654	151	293.1	272	46 W...	C15H32O2Si	Dodecanoic acid, TMS derivative
⊞ 13	replib	908	908	722	922	44	1542	263	292.8	256	33 W...	C14H28O2Si	10-Undecenoic acid, TMS derivative
14	mainlib	907	907	746	924	70	1314	491	298.7	230	2 GM	C12H26O2Si	5-Methyloctanoic acid, TMS
15	mainlib	907	907	725	923	84	1229	576	296.6	216	18 C...	C11H24O2Si	6-Methylheptanoic acid, TMS
16	mainlib	906	906	730	927	42	1558*	247	292.6	258	28 W...	C14H30O2Si	Undecanoic acid, TMS derivative
⊞ 17	mainlib	901	901	749	920	-28	2049	-244	293.8	328	47 W...	C19H40O2Si	Palmitic Acid, TMS derivative

Names / Structures — Hit List

S5a

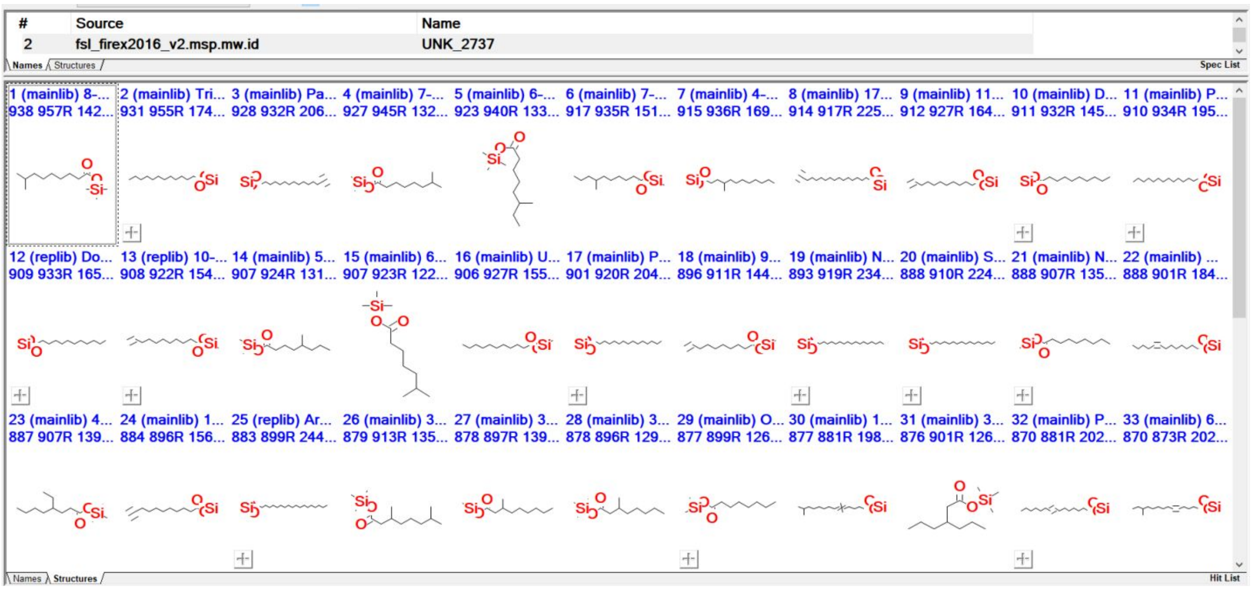

S5b

Figure S5 a,b. Hybrid search results for a member of a common class of compounds – TMS derivatives of acyclic alkyl acids. S5a) shows high scoring hit list of densely packed high scores, data and S5b) shows their chemical structures.

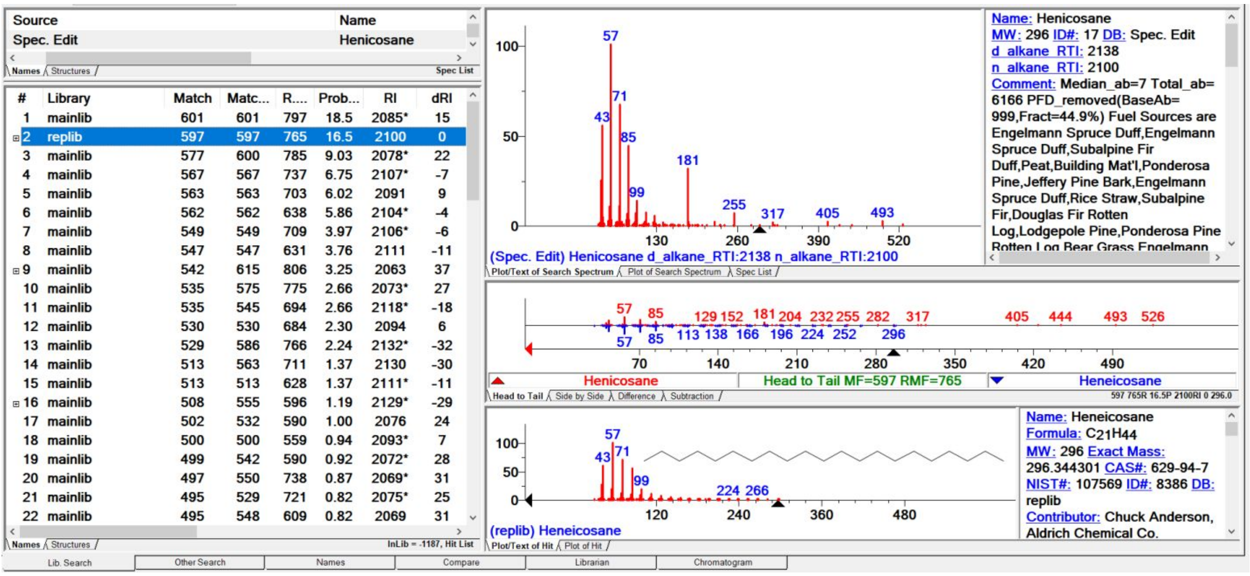
Name: Henicosane
MW: 296 ID#: 17 DB: Spec. Edit
d_alkane_RTI: 2138
n_alkane_RTI: 2100
(Spec. Edit) Henicosane d_alkane_RTI:2138 n_alkane_RTI:2100
Henicosane
Head to Tail MF=597 RMF=765
Heneicosane
Name: Heneicosane
Formula: C21H44
MW: 296 Exact Mass: 296.344301 CAS#: 629-94-7
NIST#: 107569 ID#: 8386 DB: replib
Contributor: Chuck Anderson, Aldrich Chemical Co.
(replib) Heneicosane

S6a.

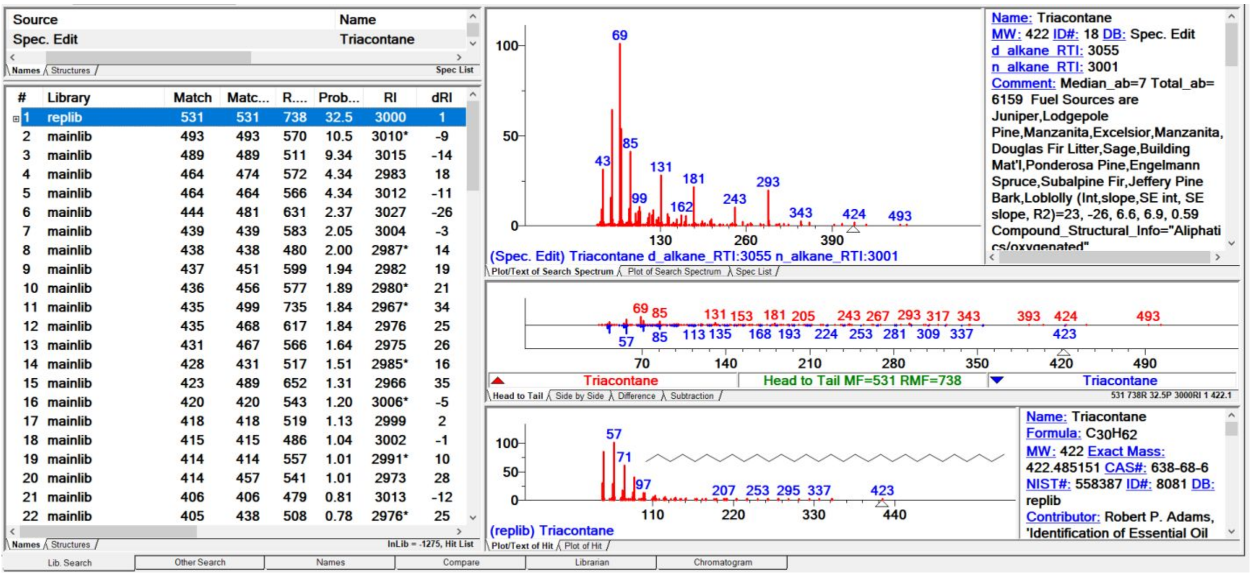
Name: Triacontane
MW: 422 ID#: 18 DB: Spec. Edit
d_alkane_RTI: 3055
n_alkane_RTI: 3001
(Spec. Edit) Triacontane d_alkane_RTI:3055 n_alkane_RTI:3001
Triacontane
Head to Tail MF=531 RMF=738
Triacontane
Name: Triacontane
Formula: C30H62
MW: 422 Exact Mass: 422.485151 CAS#: 638-68-6
NIST#: 558387 ID#: 8081 DB: replib
Contributor: Robert P. Adams, 'Identification of Essential Oil
(replib) Triacontane

S6b.

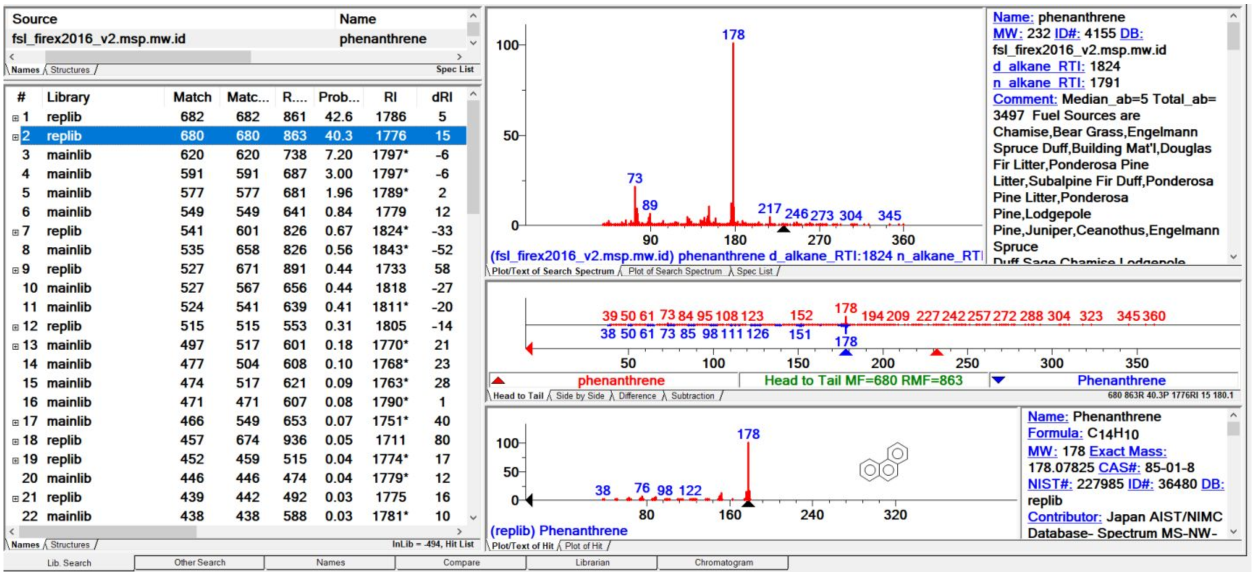

S6c.

Figure S6a-c. Low scoring RI-corrected Identity search identifications for FIREX spectra showing reduced scores due to spectrum contamination. The highlighted entries had highest corrected score.

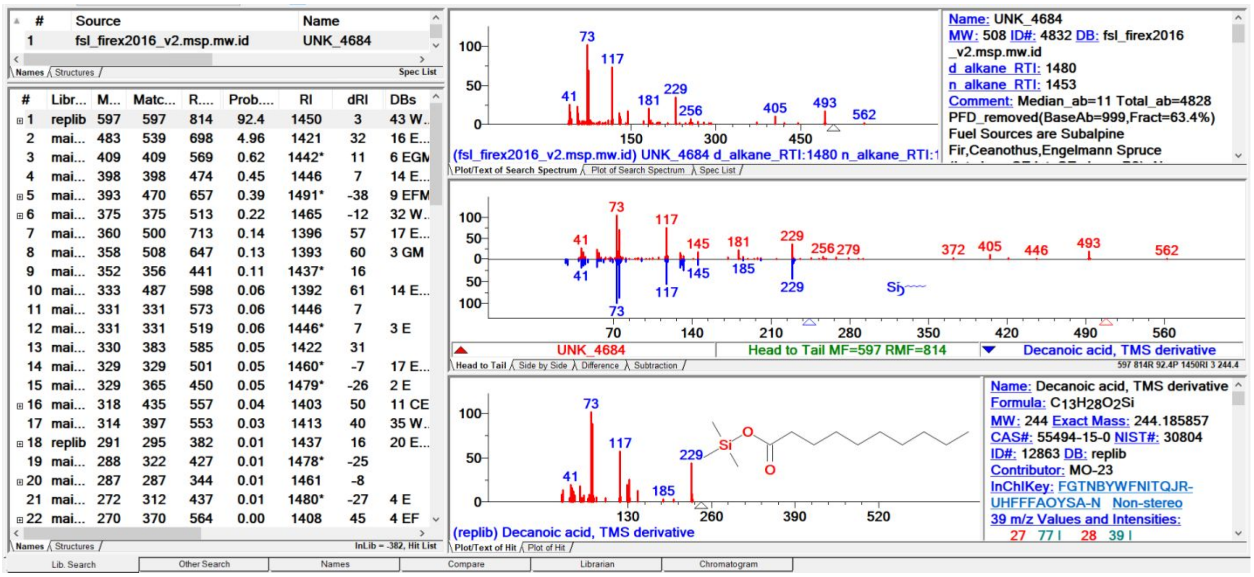

Figure S7. Despite its low score of 597 by RI-corrected Identity scoring, the spectrum UNK_4684, is very likely correctly identified in FIREX as Decanoic acid, TMS, due to its uniqueness (Probability of 92.4%), Ubiquity (otherDBs/CUI 43) and Reverse Score of 814. It is assigned a combined score of 830 (see text). Contaminant peaks, especially at high mass, are responsible for its low uncorrected score.
